# Maternal Immune Activation Affects Hippocampal Excitatory and Inhibitory Synaptic Transmission in Offspring From an Early Developmental Period to Adulthood

**DOI:** 10.3389/fncel.2020.00241

**Published:** 2020-08-04

**Authors:** Keiju Nakagawa, Hiroki Yoshino, Yoichi Ogawa, Kazuhiko Yamamuro, Sohei Kimoto, Yoshinobu Noriyama, Manabu Makinodan, Masayuki Yamashita, Yasuhiko Saito, Toshifumi Kishimoto

**Affiliations:** ^1^Department of Psychiatry, Nara Medical University, Kashihara, Japan; ^2^Department of Neurophysiology, Nara Medical University, Kashihara, Japan; ^3^Center for Medical Science, International University of Health and Welfare, Otawara, Japan

**Keywords:** CA1, synaptic transmission, maternal infection, poly I:C, schizophrenia

## Abstract

One of the risk factors for schizophrenia is maternal infection. We have previously shown that Polyriboinosinic-polyribocytidylic acid (poly I:C) induced maternal immune activation in mice caused histological changes in the hippocampal CA1 area of offspring during the developmental period and impaired sensorimotor gating in offspring during adulthood, resulting in behavioral changes. However, it remains unclear how maternal immune activation functionally impacts the hippocampal neuronal activity of offspring. We studied the effect of prenatal poly I:C treatment on synaptic transmission of hippocampal CA1 pyramidal cells in postnatal and adult offspring. Treatment with poly I:C diminished excitatory and enhanced inhibitory (GABAergic) synaptic transmission on pyramidal cells in adult offspring. During the early developmental period, we still observed that treatment with poly I:C decreased excitatory synaptic transmission and potentially increased GABAergic synaptic transmission, which was uncovered under a condition of high extracellular potassium-activated neurons. In conclusion, we demonstrate that maternal immune activation decreased excitatory and increased inhibitory synaptic transmission on hippocampal pyramidal cells from an early developmental period to adulthood, which could result in net inhibition in conjunction with poor functional organization and integration of hippocampal circuits.

## Introduction

Epidemiological studies have indicated that maternal infection is a risk factor for neurodevelopmental disorders such as schizophrenia (Brown, 2012). Several animal studies have shown that prenatal infection affects brain development by triggering the expression of proinflammatory cytokines and yields behavioral, pharmacological, and histological abnormalities that are reminiscent of schizophrenia (Deverman and Patterson, 2009; Meyer, 2014). Polyriboinosinic-polyribocytidylic acid (poly I:C), a synthetic double-stranded RNA, is a viral mimetic agent that induces a nonspecific maternal anti-virus response with a time window and has been used in animal studies of prenatal immune activation (Shi et al., 2003; Smith et al., 2007; Makinodan et al., 2008; Meyer, 2014).

The hippocampus has been implicated in the pathophysiology of schizophrenia. In particular, the hippocampal CA1 area is known to be affected at an early stage of the disease (Narr et al., 2004; Schobel et al., 2009). Decreased hippocampal volume, selective decrease in hippocampal neurons, and decreased expression of proteins and genes associated with GABAergic neurons, glutamatergic neurons, and synaptic organization in the hippocampus are observed in schizophrenia (Heckers and Konradi, 2010, 2015).

Our previous study showed that maternal poly I:C treatment in mice delayed myelination of the hippocampus in offspring in the early postnatal period and impaired sensorimotor gating as measured by prepulse inhibition (PPI) in adulthood (Makinodan et al., 2008). Hypomyelination in the early postnatal period may be linked to altered development of excitatory synaptic transmission from axon terminals (Wake et al., 2011; Gibson et al., 2014; Fields, 2015). Subsequent abnormalities in adult hippocampal function may underlie schizophrenia-like behavior.

Neural dysfunction in the hippocampus in the early postnatal period impacts the development and maturation of several brain regions, including the prefrontal cortex. Animals with Neonatal ventral hippocampal lesions (NVHLs) develop abnormal behaviors relevant to schizophrenia (including PPI), which depend on the prefrontal cortex (Tseng et al., 2009; O’Donnell, 2012). In addition to the possibility that adult hippocampal dysfunction directly underlies schizophrenia-like behavior in adulthood, maternal immune activation may disturb neonatal hippocampal function and result in abnormal behaviors after adolescence.

This study had two aims. First, we used the whole-cell patch-clamp recording to investigate whether maternal immune activation functionally affects neuronal circuits in the hippocampus of offspring in adulthood by recording excitatory and inhibitory synaptic currents in acute hippocampal slices of adult offspring from poly I:C-treated mouse dams. Second, we investigated whether the changes we observed in adult offspring became apparent in adulthood, or if their onset was earlier in development, by recording excitatory and inhibitory synaptic currents in hippocampal slices from neonatal offspring.

## Materials and Methods

### Animals

C57BL/6 mice (Japan SLC, Hamamatsu, Japan) were housed under controlled temperature and humidity, under a 12 h light/dark cycle (lights on from 8:00 AM to 8:00 PM). Food and water were available *ad libitum* throughout the experiments. Mice were mated overnight at about 3 months of age, and the day of copulation was defined as embryonic day 0 (ED0). Poly I:C was administered at a dose of 20 mg/kg to pregnant mice once on ED 12.5 (Smith et al., 2007; Makinodan et al., 2009; Ito et al., 2010). Poly I:C was dissolved in phosphate-buffered saline (PBS) at a concentration of 2 mg/ml, and 0.2 ml per 20 g body weight of poly I:C solution was injected intraperitoneally. An equivalent volume of PBS was administered to pregnant mice in the control group. Offspring mice were weaned on postnatal day 21 (PD21). Whole-cell patch-clamp recordings were performed in cornu ammonis (CA) 1 of the hippocampus during the postnatal period (PD0-4, 5–9, and 10–15) and adulthood (PD49-70) of male offspring mice to avoid sexually dimorphic effects. We used 52 male offspring mice from 32 dams in the PBS group and 56 male offspring mice from 37 dams in Poly I:C group. All experiments were approved by the animal care and use committee of Nara Medical University and conducted according to its guidelines. All efforts were made to minimize the number of animals and their suffering.

### Slice Preparation

Offspring mice were anesthetized by isoflurane and decapitated. The brains were removed and rapidly immersed in an ice-cold solution composed of (in mM): sucrose 230, KCl 2.5, NaHCO_3_ 25, NaH_2_PO_4_ 1.25, CaCl_2_ 0.5, MgSO_4_ 10, and D-glucose 11; pH 7.4. Horizontal slices (350 μm) of the hippocampus were cut in this solution with a vibrating tissue slicer (Vibratome 1,000 Plus 102, Pelco International, Redding, CA, USA) and incubated in standard artificial cerebrospinal fluid (ACSF, composition in mM: NaCl 125, KCl 2.5, NaHCO_3_ 25, NaH_2_PO_4_ 1.25, CaCl_2_ 2.0, MgCl_2_ 1.0, and D-glucose 25; pH 7.4) bubbled with 95% O_2_ / 5% CO_2_ at 32°C for at least 1 h.

### Voltage-Clamp Recording

After incubation, slices were transferred to a recording chamber (RC-25F, volume, 150 μl, Warner Instrument/Harvard Apparatus, Holliston, MA, USA), which was mounted on a fixed stage of an upright microscope (BX50WI, Olympus, Tokyo, Japan). The recording chamber was continuously perfused with oxygenated ACSF at a rate of 2.0 ml/min. Whole-cell patch-clamp recordings were performed on pyramidal neurons of hippocampal CA1 slices at 31–33°C with a patch-clamp amplifier (EPC 9, Heka, Lambrecht, Germany). We recorded one neuron from each brain slice. Each cell was identified morphologically by its shape and location with an infrared CCD camera (C2741–79, Hamamatsu Photonics, Hamamatsu, Japan) through an objective lens (LUMPlanFL40xW/IR2, N.A., 0.80, W.D., 3.3 mm, Olympus). Patch pipettes were prepared from borosilicate glass capillary tubes using a two-stage vertical puller (PP-830, Narishige, Tokyo, Japan; Pineda et al., 2013). Pipettes were filled with a low-chloride pipette solution containing (in mM): K gluconate 141, KCl 4.0, MgCl_2_ 2.0, HEPES 10, Mg-ATP 2.0, Na-GTP 0.3, and EGTA 0.2 for the recording of glutamatergic synaptic currents; and with a high-chloride pipette solution containing (in mM): K gluconate 95, KCl 50, MgCl_2_ 2.0, HEPES 10, Mg-ATP 2.0, Na-GTP 0.3, and EGTA 0.2 for the recording of GABAergic synaptic currents; pH was adjusted to 7.2 with KOH in both cases. The resistance of the recording electrode was adjusted to 3–4 MΩ. Series resistance was compensated by 60–70% and continuously monitored throughout the experiments. When the series resistance increased by more than 20%, the recording was discarded. Signals were digitized at 5 kHz for recording postsynaptic currents (PSCs; Noriyama et al., 2006). Spontaneous GABA_A_ receptor-mediated postsynaptic currents (sGABA_A_-PSCs) were recorded in the presence of the non-NMDA receptor blocker 6-cyano-7-nitroquinoxaline-2,3dione (CNQX; 10 μM) and the NMDA receptor blocker MK-801 (10 μM). The cell was voltage-clamped at −70 mV, at which membrane potential GABA_A_-PSCs appeared as inward currents. Miniature GABA_A_-PSCs (mGABA_A_-PSCs) were recorded in the presence of CNQX (10 μM), MK-801 (10 μM), and the selective inhibitor of voltage-gated sodium channels tetrodotoxin (TTX; 1 μM), to block voltage-dependent sodium channels at −70 mV. The mGABA_A_-PSCs were abolished by the application of the GABA_A_ receptor antagonist bicuculline (10 μM). Spontaneous excitatory postsynaptic currents (sEPSCs) were recorded with pipettes filled with low-chloride pipette solution from the cell with voltage-clamp at −70 mV, at which membrane potential EPSCs and GABA_A_-PSCs were distinguishable because EPSCs appeared as inward currents. Miniature EPSCs (mEPSCs) were recorded in the presence of TTX (1 μM) to block voltage-dependent sodium channels at −70 mV. sEPSCs were recorded without TTX.

### Current-Clamp Recording

For the current-clamp recordings, series resistance was monitored and canceled using a bridge circuit, and pipette capacitance were compensated. Voltage signals were low-pass filtered at 10 kHz and digitized at 20 kHz. The baseline membrane potential was maintained near −70 mV with current injection. To examine action potential and subthreshold membrane properties, we recorded membrane potential responses to hyperpolarizing and depolarizing current pulses (500 ms in duration; Yamamuro et al., 2018).

### Normal and High Extracellular Potassium Conditions

Depolarization induced by the application of solutions containing high potassium is one of the most frequently used stimuli to evoke the exocytotic release of neurotransmitters (Rodríguez-Navarro et al., 2009). In the present study, the extracellular potassium concentration was increased from 2.5 mM (normal potassium condition) to 7.5 mM (high potassium condition) to enhance synaptic activity.

### Data Analysis

Values are presented as mean ± SEM. and the number of cells tested (*n*). Unpaired Student *t*-test with Welch correction for non-equal variance was used to compare data between the PBS and poly I:C groups. Two-way ANOVA was used to compare data from the three different developmental periods. Statistical significance was set at the level of *P* < 0.05.

## Results

### Prenatal Poly I:C Treatment Reduced Excitatory Synaptic Inputs Onto CA1 Pyramidal Cells in Adulthood

**Figure 1:**
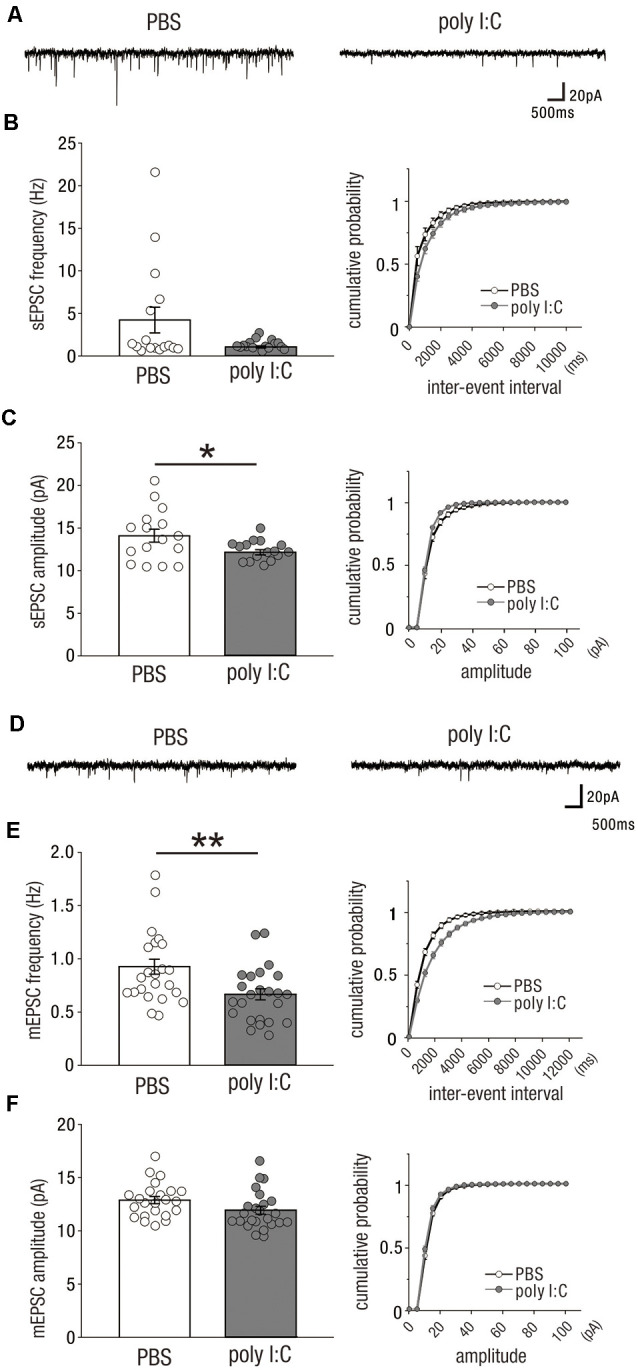
Prenatal poly I:C treatment reduces excitatory synaptic inputs onto CA1 pyramidal cells in adulthood. **(A)** Representative data of spontaneous excitatory postsynaptic currents (sEPSCs) recorded from a CA1 pyramidal cell of a PBS-treated mouse (left) and poly I:C-treated mouse (right). **(B)** Left: the frequency of sEPSCs of poly I:C-treated mice showed a trend to be significantly lower than that of PBS-treated mice. Right: Cumulative probability of sEPSC frequencies shows a right-skewed curve in poly I:C-treated mice compared to that in PBS-treated mice. **(C)** Left: the amplitude of sEPSCs of poly I:C-treated mice was significantly smaller than that of PBS-treated mice. Right: cumulative probability of sEPSC amplitudes shows a left-skewed curve in poly I:C-treated mice compared to that in PBS-treated mice. **(D)** Representative data of miniature excitatory postsynaptic currents (mEPSCs) recorded from a CA1 pyramidal cell of a PBS-treated mouse (left) and poly I:C-treated mouse (right). **(E)** Left: the frequency of mEPSCs in poly I:C-treated mice was significantly lower than that in PBS-treated mice. Right: cumulative probability of mEPSC frequencies shows a right-skewed curve in poly I:C-treated mice compared to that in PBS-treated mice. **(F)** Left: there was no significant difference in mEPSC amplitude between PBS-treated and poly I:C-treated mice. Right: overlapping cumulative probability curve between PBS-treated and poly I:C-treated mice, *p < 0.05, **p < 0.01. Data are presented as means, and error bars indicate SEM. Poly I:C, polyriboinosinic-polyribocytidilic acid; PBS, phosphate-buffered saline.

In our previous study, we found that prenatal poly I:C treatment reduced mRNA and protein levels of myelin basic protein (MBP), and the diameter of axonal segments in the CA1 area of the hippocampus in the early postnatal period, which delayed myelination and caused behaviorally impaired PPI in adulthood (Makinodan et al., 2008). As hippocampal lesions impair sensorimotor processing as measured by the PPI test (Bast and Feldon, 2003), delayed myelination in the early postnatal period may impact adult hippocampal function. Myelination is a crucial mechanism that determines conduction velocity to modify neuronal connectivity of neural circuits by optimizing the timing of action potential arrival (Fields, 2015). As such, delayed myelination in this period could affect the activity-dependent development of functional excitatory synapses onto CA1 pyramidal cells. To test this possibility, we recorded sEPSCs (Figure 1A) and mEPSCs (Figure 1D) from CA1 pyramidal cells in the offspring of poly I:C-treated and PBS-treated dams during adulthood (PD49–70).

A significant reduction in the amplitude of sEPSCs was observed in the offspring of poly I:C-treated dams compared to that in the offspring of PBS-treated dams (Figure 1C, left; sEPSC amplitude, PBS: 14.10 ± 0.76 pA, *n* = 16 from three animals; poly I:C: 12.23 ± 0.29 pA, *n* = 16 from three animals, −13.3% of PBS; Student *t*-test: *P* < 0.05). There was a trend towards a significant difference in sEPSC frequency between both groups of mice (Figure 1B, left; sEPSC frequency, PBS: 4.23 ± 1.50 Hz, *n* = 16 from 3 animals; poly I:C: 1.07 ± 0.13 Hz, *n* = 16 from three animals, −74.7% of PBS; Student *t*-test: *P* = 0.054).

We also analyzed the mEPSCs of CA1 pyramidal cells. mEPSCs are resistant to TTX and represent the excitation-independent quantal transmitter release onto the recorded neuron. mEPSC frequency was significantly reduced by poly I:C-treatment compared to that of the control group (Figure 1E, left; mEPSC frequency, PBS: 0.92 ± 0.07 Hz, *n* = 23 from five animals; poly I:C: 0.67 ± 0.05 Hz, *n* = 24 from five animals, −27.2% of PBS; Student *t*-test: *P* < 0.01). Poly I:C treatment showed a trend towards having a significant effect on mEPSC amplitude (Figure 1F, left; mEPSC amplitude, PBS: 12.89 ± 0.33 pA, *n* = 23 from five animals; poly I:C: 11.94 ± 0.37 pA, *n* = 24 from five animals, −7.4% of PBS; Student *t*-test: *P* = 0.06). These results suggest that prenatal poly I:C treatment decreases excitatory synaptic inputs onto CA1 pyramidal cells in adult offspring.

### Prenatal Poly I:C Treatment Increased Inhibitory (GABAergic) Drive Onto CA1 Pyramidal Cells in Adulthood

Previous studies have suggested that the development of GABAergic interneurons and their synaptic activities are vulnerable to prenatal infection and/or inflammation (Nyffeler et al., 2006; Samuelsson et al., 2006; Meyer et al., 2008). In the offspring of poly I:C-treated dams, the inhibitory drive of CA1 neuronal circuits may be reduced to compensate for the reduced excitatory synaptic inputs onto pyramidal cells as shown in Figure 1; alternatively, prenatal poly I:C treatment may completely reduce neuronal circuit activity of the CA1 to increase inhibitory synaptic inputs in parallel with the reduced excitatory synaptic inputs.

**Figure 2:**
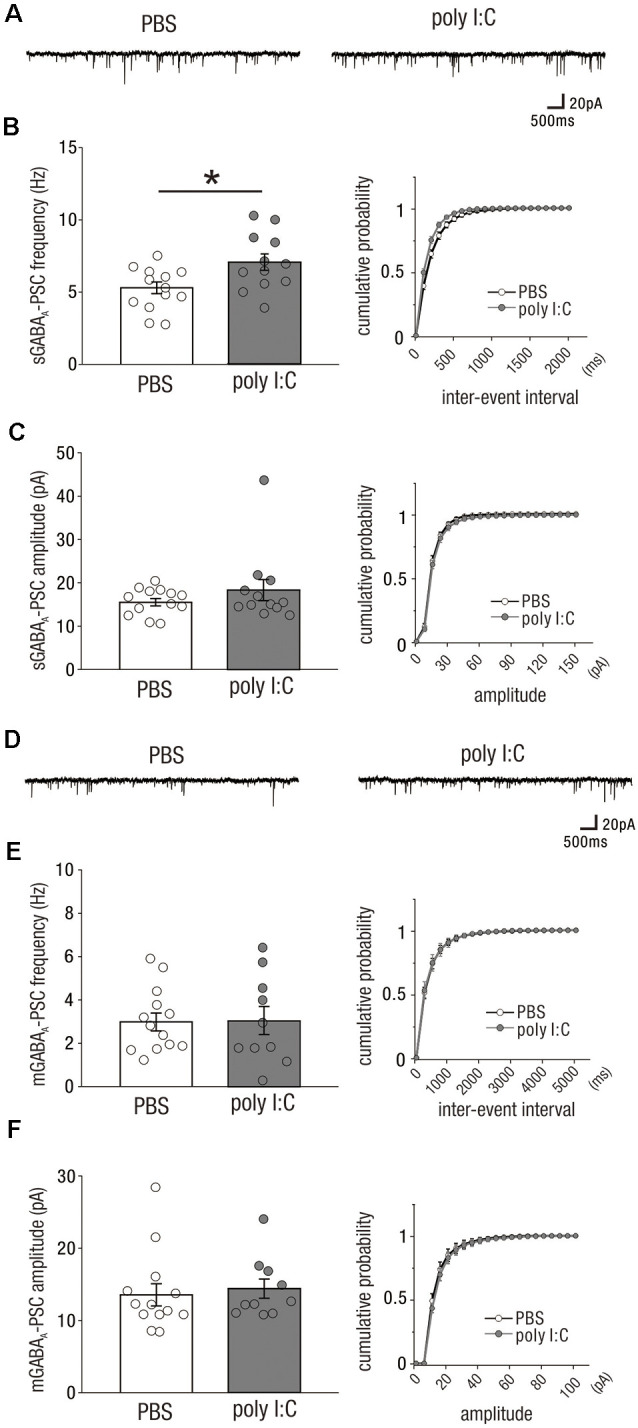
Prenatal poly I:C treatment increases inhibitory (GABAergic) drive onto CA1 pyramidal cells in adulthood. **(A)** Representative data of spontaneous GABA_A_ receptor-mediated postsynaptic currents (sGABA_A_-PSCs) recorded from a CA1 pyramidal cell of a PBS-treated mouse (left) and poly I:C-treated mouse (right). **(B)** Left: the frequency of sGABA_A_-PSCs of poly I:C treated-mice was significantly higher than that of PBS-treated mice. Right: cumulative probability of sGABA_A_-PSC frequencies shows a left-skewed curve in poly I:C-treated mice compared to that in PBS-treated mice. **(C)** Left: there was no significant difference in sGABA_A_-PSC amplitudes between PBS-treated and poly I:C-treated mice. Right: Overlapping cumulative probability curve between PBS-treated and poly I:C-treated mice. **(D)** Representative data of miniature GABA_A_ receptor-mediated postsynaptic currents (mGABA_A_-PSCs) recorded from a CA1 pyramidal cell of a PBS-treated mouse (left) and poly I:C-treated mouse (right). **(E,F)** There were no significant differences in mGABA_A_-PSC frequencies and amplitudes, *p < 0.05. Data are presented as means, and error bars indicate SEM. Poly I:C, polyriboinosinic-polyribocytidilic acid; PBS, phosphate-buffered saline.

To test these possibilities, we recorded spontaneous and miniature inhibitory (GABAergic) postsynaptic currents [sGABA_A_-PSCs (Figure 2A) and mGABA_A_-PSCs (Figure 2D)] from CA1 pyramidal cells of both adult offspring of PBS-treated and poly I:C-treated dams. The frequency of sGABA_A_-PSCs in the offspring of poly I:C-treated dams were significantly higher than that in the offspring of PBS-treated dams (Figure 2B, left; sGABA_A_-PSCs frequency, PBS: 5.30 ± 0.41 Hz, *n* = 13 from six animals; poly I:C: 7.08 ± 0.57 Hz, *n* = 12 from five animals, +33.6% of PBS; Student *t*-test: *P* < 0.05). There was no significant difference in the amplitude of sGABA_A_-PSCs between the offspring of poly I:C- and PBS-treated dams (Figure 2C, left; sGABA_A_-PSC amplitude, PBS: 15.53 ± 0.86 pA, *n* = 13 from six animals; poly I:C: 18.47 ± 2.44 pA, *n* = 12 from five animals, Student *t*-test: *P* = 0.28).

We analyzed mGABA_A_-PSCs, and found no significant differences in their frequency and amplitude between the offspring of poly I:C- and PBS-treated dams (Figure 2E, left; mGABA_A_-PSC frequency, PBS: 2.99 ± 0.41 Hz, *n* = 13 from six animals; poly I:C: 3.05 ± 0.65, *n* = 10 from five animals, Student *t*-test: *P* = 0.93; Figure 2F, left; mGABA_A_ amplitude, PBS: 13.54 ± 1.54 pA, *n* = 13 from six animals; poly I:C: 14.41 ± 1.32 pA, *n* = 10 from five animals, Student *t*-test: *P* = 0.68). These results suggest that prenatal poly I:C treatment increases the frequency of action potential-dependent GABAergic synaptic inputs onto CA1 pyramidal cells in adult offspring.

### The Impact of Prenatal Poly I:C Treatment on Excitatory Drive Onto CA1 Pyramidal Cells Emerged From an Early Postnatal Period

**Figure 3:**
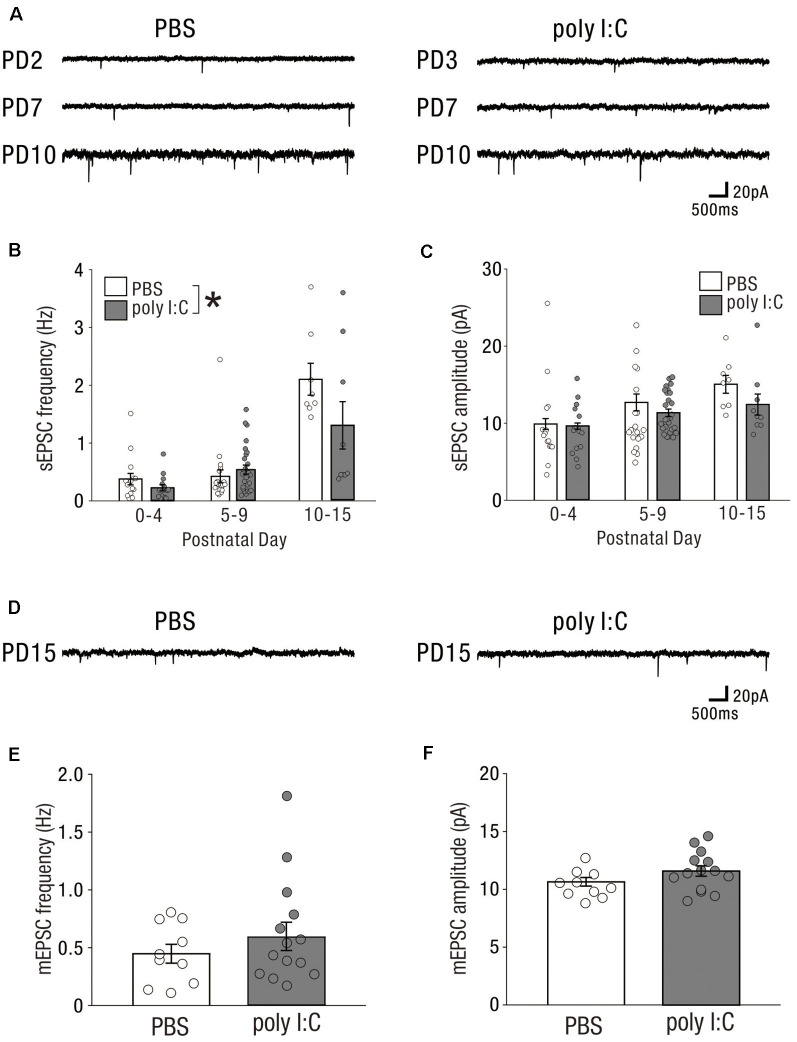
In the early postnatal period, prenatal poly I:C treatment partially affects excitatory drive onto CA1 pyramidal cells. **(A)** Representative data of sEPSCs recorded from a CA1 pyramidal cell of a PBS-treated mouse (left) and poly I:C-treated mouse (right) in each PD during the neonatal period. **(B)** There was a significant difference in sEPSC frequency between PBS-treated and poly I:C-treated mice. **(C)** There was no significant difference in sEPSC amplitude between PBS-treated and poly I:C mice. **(D)** Representative data of mEPSCs recorded from a CA1 pyramidal cell of a PBS-treated mouse (left) and poly I:C-treated mouse (right) in each PD during the neonatal period. **(E,F)** There were no significant differences in mEPSC frequency and amplitude between PBS-treated and poly I:C-treated mice, *p < 0.05. Data are presented as means, and error bars indicate SEM. Poly I:C, polyriboinosinic-polyribocytidilic acid; PBS, phosphate-buffered saline; sEPSC, spontaneous excitatory postsynaptic current; mEPSC, miniature excitatory postsynaptic current.

Prenatal poly I:C treatment has been shown to impair measures of PPI (Shi et al., 2003; Ozawa et al., 2006; Smith et al., 2007; Makinodan et al., 2008; Meyer et al., 2008), disrupt latent inhibition (Meyer et al., 2006, 2008; Smith et al., 2007), increase amphetamine- methamphetamine- and MK-801-induced locomotion (Ozawa et al., 2006; Meyer et al., 2008), and reduce social interaction (Shi et al., 2003; Smith et al., 2007); these abnormal behaviors are relevant to schizophrenia. Similarly, rodents with NVHLs have been shown to develop abnormal behaviors relevant to schizophrenia (Lipska et al., 1993, 1995; Tseng et al., 2009). Maternal infection may perturb neonatal hippocampal function to induce abnormal behavior after adolescence. To examine whether prenatal poly I:C treatment impaired neuronal activity in the neonatal hippocampus, we recorded sEPSCs (Figure 3A) and mEPSCs (Figure 3D) from CA1 pyramidal cells at the following three periods: postnatal day (PD) 0–4, PD5–9, and PD10–15.

The frequencies of sEPSCs in offspring of PBS-treated dams were 0.38 ± 0.10 Hz (PD0–4: *n* = 15 from three animals), 0.42 ± 0.11 Hz (PD5–9: *n* = 20 from four animals), and 2.09 ± 0.28 Hz (PD10–15: *n* = 8 from two animals); whereas those in offspring of poly I:C-treated dams were 0.23 ± 0.05 Hz (PD0–4: *n* = 15 from three animals), 0.54 ± 0.08 Hz (PD5–9: *n* = 27 from five animals), and 1.30 ± 0.41 Hz (PD10–15: *n* = 9 from two animals). Two-way ANOVA demonstrated a significant difference in sEPSC frequency between PBS-treated and poly I:C-treated mice (Figure 3B, PBS: 0.72 ± 0.07 Hz, *n* = 43 from nine animals; poly I:C: 0.58 ± 0.10 Hz, *n* = 51 from 10 animals, −19.4% of PBS, effect of treatment: *F*_(1,88)_ = 4.72, *P* < 0.05) and a significant PD × treatment interaction (Figure 3B, interaction: *F*_(2,88)_ = 4.05, *P* < 0.05), although *post hoc* tests did not show any significant differences between PBS-treated and poly I:C-treated mice at any developmental period. The amplitude of sEPSCs in offspring of PBS-treated dams was 9.90 ± 0.70 pA (PD0–4: *n* = 15 from three animals), 12.68 ± 1.09 pA (PD5–9: *n* = 20 from four animals), and 15.05 ± 1.16 pA (PD10–15: *n* = 8 from two animals), whereas that in offspring of poly I:C-treated dams was 9.64 ± 0.41 pA (PD0–4: *n* = 15 from three animals), 11.34 ± 0.50 (PD5–9: *n* = 27 from five animals), and 12.49 ± 1.37 pA (PD10–15: *n* = 9 from two animals). Two-way ANOVA demonstrated no significant difference in the amplitude of sEPSCs between offspring of PBS-treated and poly I:C-treated dams (Figure 3C, effect of treatment: *F*_(1,88)_ = 3.43, *P* = 0.07).

We recorded mEPSCs only on PD10–15, as on PD0–9 too few mEPSCs were detectable under our recording conditions. On PD10–15, there were no significant differences in both frequency and amplitude of mEPSCs between offspring of PBS-treated and poly I:C-treated dams (Figure 3E, mEPSC frequency, PBS: 0.45 ± 0.08 Hz, *n* = 10 from five animals, poly I:C: 0.59 ± 0.12 Hz, *n* = 14 from five animals, Student *t*-test: *P* = 0.33; Figure 3F, mEPSC amplitude, PBS: 10.65 ± 0.37 pA, *n* = 10 from five animals, poly I:C: 11.58 ± 0.45 pA, *n* = 14 from five animals, Student *t*-test: *P* = 0.12). These results suggest that prenatal poly I:C treatment already has an impact on the excitatory drive at an early postnatal stage, as the reduction in sEPSC frequency emerges.

### Lack of Effect of Poly I:C Treatment on GABAergic Inhibitory Drive or GABAergic Synaptic Inputs in the Early Postnatal Period

**Figure 4:**
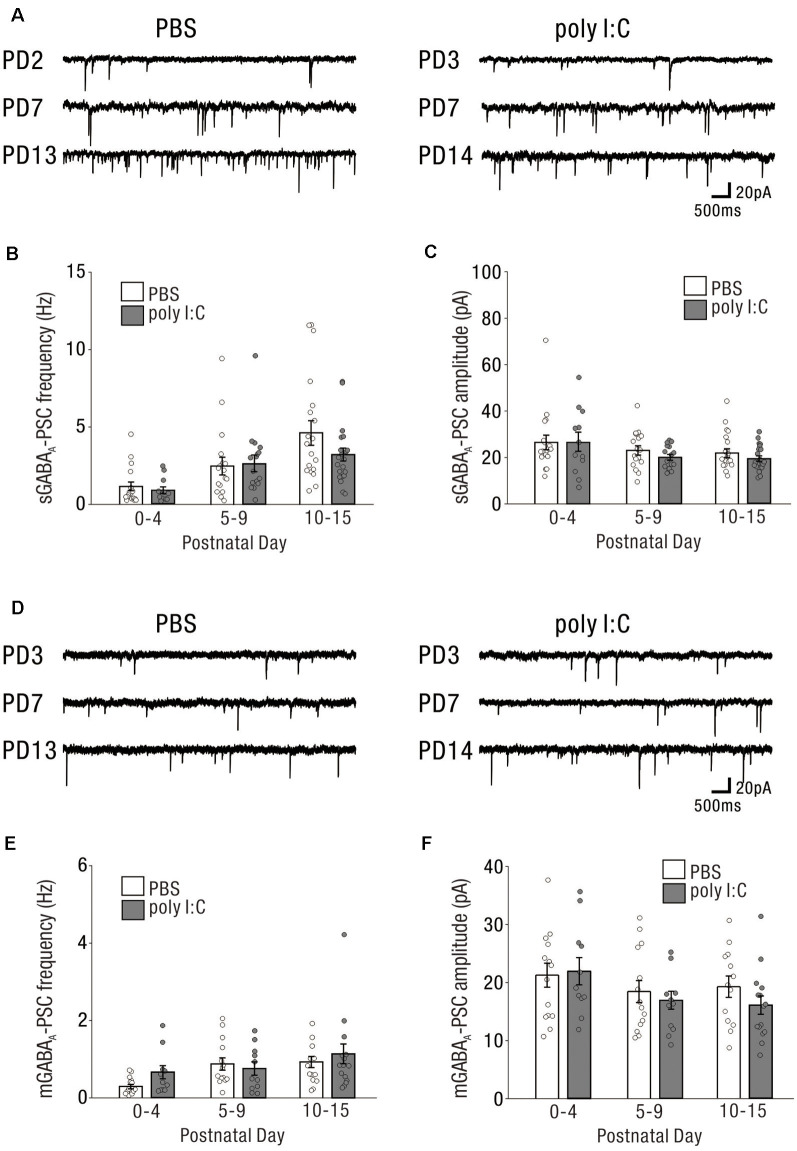
The impact on GABAergic drive and synaptic inputs onto CA1 pyramidal cells in the early postnatal period by prenatal poly I:C treatment are undetected.** (A)** Representative data of sGABA_A_-PSCs recorded from a CA1 pyramidal cell of a PBS-treated mouse (left) and poly I:C-treated mouse (right) in each PD during the neonatal period. **(B,C)** There were no significant differences in sGABA_A_-PSC frequency and amplitude between PBS-treated and poly I:C-treated mice. **(D)** Representative data of mGABA_A_-PSCs recorded from a CA1 pyramidal cell of a PBS-treated mouse (left) and poly I:C-treated mouse (right) in each PD during the neonatal period. **(E,F)** There were no significant differences in mGABA_A_-PSCs frequency and amplitude between PBS-treated and poly I:C-treated mice. Poly I:C, polyriboinosinic-polyribocytidilic acid; PBS, phosphate-buffered saline; mGABA_A_-PSC, miniature GABA_A_ receptor-mediated postsynaptic current; sGABA_A_-PSC, spontaneous GABA_A_ receptor-mediated postsynaptic current.

Similar to excitatory synaptic inputs affected by prenatal poly I:C treatment, GABAergic synaptic inputs at the early postnatal period may be altered by prenatal poly I:C treatment. We recorded sGABA-PSCs (Figure 4A) and mGABA-PSCs (Figure 4D) from CA1 pyramidal cells of neonatal offspring of both PBS-treated and poly I:C-treated dams.

The frequencies of sGABA-PSCs in offspring of PBS-treated dams were 1.16 ± 0.28 (PD0–4: *n* = 18 from nine animals), 2.52 ± 0.57 (PD5–9: *n* = 17 from eight animals), and 4.64 ± 0.79 (PD10–15: *n* = 19 from seven animals). Those in offspring of poly I:C-treated dams were 0.92 ± 0.21 (PD0–4: *n* = 12 from six animals), 2.65 ± 0.54 (PD5–9: *n* = 16 from six animals), and 3.25 ± 0.42 (PD10–15: *n* = 21 from nine animals). Two-way ANOVA revealed that there was no significant difference in these frequencies between offspring of PBS-treated and poly I:C-treated dams (Figure 4B, effect of treatment: *F*_(1,97)_ = 1.26, *P* = 0.26) and no significant PD × treatment interaction (Figure 4B, interaction: *F*_(2,97)_ = 1.16, *P* = 0.32). For the amplitudes of sGABA-PSCs, there was no significant difference (Figure 4C, effect of treatment: *F*_(1,97)_ = 0.91, *P* = 0.34) and no significant PD × treatment interaction (Figure 4C, interaction: *F*_(2,97)_ = 0.23, *P* = 0.79) between offspring of PBS-treated and poly I:C-treated dams (PBS: PD0–4: 26.47 ± 3.08 pA, *n* = 18 from nine animals; PD5–9: 23.02 ± 1.97 pA, *n* = 17 from eight animals; PD10–15: 21.95 ± 1.87 pA, *n* = 19 from seven animals; poly I:C: PD0–4: 26.53 ± 4.07 pA, *n* = 12 from six animals; PD5–9: 20.00 ± 1.25 pA, *n* = 16 from six animals; PD10–15: 19.60 ± 1.18 pA, *n* = 21 from nine animals).

For both the frequencies and amplitudes of mGABA-PSCs in neonatal periods, there were no significant differences between offspring of PBS-treated and poly I:C-treated dams, and no significant interaction (Figure 4E, mGABA_A_ frequency: effect of treatment: *F*_(1,72)_ = 1.16, *P* = 0.29, interaction: *F*_(2,72)_ = 0.97, *P* = 0.38; PBS: PD0–4: 0.29 ± 0.06 Hz, *n* = 14 from eight animals; PD5–9: 0.88 ± 0.16 Hz, *n* = 14 from eight animals; PD10–15: 0.94 ± 0.14 Hz, *n* = 13 from seven animals; poly I:C: PD0–4: 0.66 ± 0.17 Hz, *n* = 11 from five animals; PD5–9: 0.76 ± 0.17 Hz, *n* = 11 from six animals; PD10–15: 1.14 ± 0.26 Hz, *n* = 15 from nine animals, Figure 4F, mGABA_A_ amplitude: effect of treatment: *F*_(1,72)_ = 0.74, *P* = 0.39, interaction: *F*_(2,72)_ = 0.52, *P* = 0.59; PBS: PD0–4: 21.28 ± 2.07 pA, *n* = 14 from eight animals; PD5–9: 18.43 ± 1.90 pA, *n* = 14 from eight animals; PD10–15: 19.21 ± 1.84 pA, *n* = 13 from seven animals; poly I:C: PD0–4: 21.96 ± 2.36 pA, *n* = 11 from five animals; PD5–9: 16.92 ± 1.54 pA, *n* = 11 from six animals; PD10–15: 16.02 ± 1.58 pA, *n* = 15 from nine animals).

Prenatal poly I:C treatment did not affect GABAergic synaptic inputs onto neonatal CA1 pyramidal cells under the same experimental conditions in which we observed an effect of poly I:C treatment on neonatal glutamatergic transmission (Figure 3).

### High Extracellular Potassium-Induced Excitation Unmasked Potentially Aberrant GABAergic Transmission Onto Neonatal CA1 Pyramidal Cells by Prenatal Poly I:C Treatment

Immature neuronal circuit activity was generally lower than that of mature circuits (Figures 3, 4). From a methodological standpoint, background synaptic activity is lower in acute brain slices than in the intact brain (Paré et al., 1998). Further, we used a relatively low potassium concentration ([K^+^] = 2.5 mM) in ACSF in the previous recordings. The low synaptic activity induced by these factors may have obscured the effects of poly I:C treatment on GABAergic transmission onto neonatal CA1 pyramidal cells.

**Figure 5:**
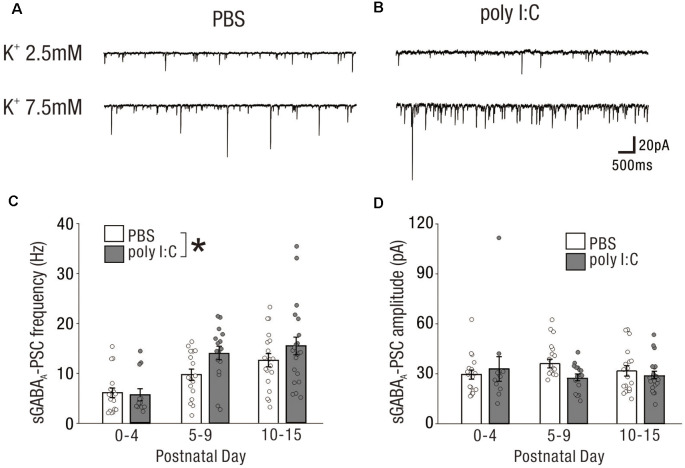
High extracellular potassium-induced excitation unmasks potentially aberrant GABAergic transmission onto neonatal CA1 pyramidal cells by prenatal poly I:C treatment. **(A,B)** Representative sGABA_A_-PSCs of a PBS-treated mouse **(A)** and poly I:C-treated mouse **(B)** in the low potassium condition (upper: [K^+^] = 2.5 mM) and high potassium condition (lower: [K^+^] = 7.5 mM). **(C,D)** In the high potassium condition, there was a significant difference in sGABA_A_-PSC frequency between PBS-treated and poly I:C-treated mice **(C)**. No significant difference in sGABA_A_-PSC amplitudes was observed between the groups, *p < 0.05. Data are presented as means, and error bars indicate SEM. Poly I:C, polyriboinosinic-polyribocytidilic acid; PBS, phosphate-buffered saline; sGABA_A_-PSC, spontaneous GABA_A_ receptor-mediated postsynaptic current.

To test this possibility, we recorded sGABA-PSCs from CA1 pyramidal cells in high potassium ACSF ([K+] = 7.5 mM) to increase synaptic activity. Representative data are shown in Figures 5A–B. The frequency of sGABA-PSCs was increased in offspring of both PBS-treated and poly I:C-treated dams in the high potassium condition ([K+] = 7.5 mM) compared to that in the normal potassium condition ([K+] = 2.5 mM).

In the high potassium condition, the frequency of sGABA-PSCs in offspring of poly I:C-treated dams was significantly higher than that in offspring of PBS-treated dams (Figure 5C, PBS: 9.53 ± 0.75 Hz, *n* = 54 from 24 animals; poly I:C: 12.61 ± 1.07 Hz, *n* = 49 from 21 animals, +32.2% of PBS, effect of treatment: *F*_(1,97)_ = 3.98, *P* < 0.05; interaction: *F*_(2,97)_ = 1.39, *P* = 0.25; PBS: PD0–4: 6.14 ± 0.94 Hz, *n* = 18 from nine animals; PD5–9: 9.72 ± 1.12 Hz, *n* = 17 from eight animals; PD10–15: 12.56 ± 1.32 Hz, *n* = 19 from seven animals; poly I:C: PD0–4: 5.74 ± 1.21 Hz, *n* = 12 from six animals; PD5–9: 14.06 ± 1.35 Hz, *n* = 16 from six animals; PD10–15: 15.44 ± 1.77 Hz, *n* = 21 from nine animals), although *post hoc* tests did not show any significant differences between PBS-treated and poly I:C-treated mice at any developmental period. There was no significant difference in the amplitude of sGABA-PSCs between PBS-treated and poly I:C-treated mice (Figure 5D, effect of treatment: *F*_(1,97)_ = 0.81, *P* = 0.37; interaction, *F*_(2,97)_ = 1.41, *P* = 0.25; PBS: PD0–4: 29.55 ± 2.76 pA, *n* = 18 from nine animals; PD5–9: 35.93 ± 2.62 pA, *n* = 17 from eight animals; PD10–15: 31.58 ± 3.05 pA, *n* = 19 from seven animals; poly I:C: PD0–4: 32.90 ± 7.41, *n* = 12 from six animals; PD5–9: 27.73 ± 2.04 pA, *n* = 16 from six animals; PD10–15: 29.14 ± 2.20 pA, *n* = 21 from nine animals). These results suggest that prenatal poly I:C treatment also affects GABAergic transmission onto neonatal CA1 pyramidal cells.

### Prenatal Poly I:C Treatment Lowered the Action Potential Threshold of Pyramidal Cells in the Neonatal Period

**Figure 6:**
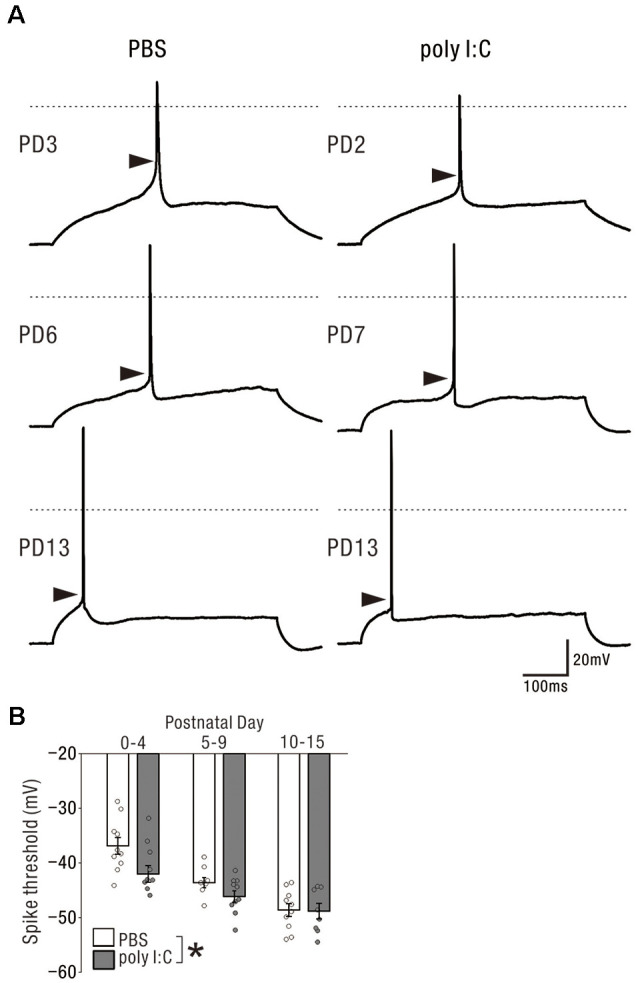
Prenatal poly I:C treatment lowered the action potential threshold of pyramidal cells in the neonatal period. **(A)** Representative traces showing action potentials elicited by the rheobase current injection. Each arrow head indicates the estimated threshold voltage for action potential generation. In each traces, 0 mV level in membrane potential is shown as a horizontal dotted line. **(B)** There was a significant difference in action potential threshold between PBS-treated and poly I:C-treated mice, *p < 0.05. Data are presented as means, and error bars indicate SEM. Poly I:C, polyriboinosinic-polyribocytidilic acid; PBS, phosphate-buffered saline.

Finally, we compared the membrane properties of pyramidal cells between offspring from PBS-treated and poly I:C-treated dams (Table 1, Figure 6A) by performing current-clamp recording. The poly I:C treatment group had a significantly lower action potential threshold than that of the PBS group in the neonatal period (Figure 6B, PBS: −43.00 ± 1.19 mV, *n* = 28 from 23 animals; poly I:C: −45.55 ± 0.91 mV, *n* = 27 from 22 animals, effect of treatment: *F*_(1. 49)_ = 6.00, *P* < 0.05; interaction: *F*_(2. 49)_ = 1.79, *P* = 0.17; PBS: PD0–4: −36.87 ± 1.53 mV, *n* = 10 from eight animals; PD5–9: −43.61 ± 0.94 mV, *n* = 8 from seven animals; PD10–15: −48.61 ± 1.16 mV, *n* = 10 from eight animals; poly I:C: PD0–4: −42 ± 1.54 mV, *n* = 9 from six animals; PD5–9: −46.14 ± 1.04 mV, *n* = 10 from eight animals; PD10–15: −48.81 ± 1.41 mV, *n* = 8 from eight animals), but not in adulthood, although *post hoc* tests did not show any significant differences between PBS-treated and poly I:C-treated mice at any developmental period. There was no statistically significant difference in action potential amplitude and afterhyperpolarization amplitude between PBS-treated and poly I:C-treated groups in both the neonatal period and adulthood. Table 2 summarizes the comparative data of EPSCs and GABA-PSCs between the PBS-treated and poly I:C-treated groups in the neonatal period and adulthood.

**Table 1:** Membrane properties of pyramidal cells from offspring of PBS-treated and poly I:C-treated dams.

	Postnatal day	PBS (*n*)	Poly I:C (*n*)	
Action potential threshold (mV)	0–4	−36.87 ± 1.53 (10)	−42.00 ± 1.54 (9)	*F*_(1.49)_ = 6.00
				*P* < 0.05^a^
	5–9	−43.61 ± 0.94 (8)	−46.14 ± 1.04 (10)
	10–15	−48.61 ± 1.16 (10)	−48.81 ± 1.41 (8)
	adult	−50.08 ± 1.15 (12)	−49.28 ± 0.94 (16)	ns^b^
Action potential amplitude (mV)	0–4	50.95 ± 4.19 (10)	55.22 ± 5.82 (9)	ns^a^
	5–9	78.95 ± 3.04 (8)	81.19 ± 3.49 (10)
	10–15	92.75 ± 1.33 (10)	91.35 ± 2.04 (8)
	adult	92.93 ± 2.42 (12)	91.91 ± 1.69 (16)	ns^b^
Afterhyperpolarization amplitude (mV)	0–4	−17.25 ± 1.43 (10)	−17.27 ± 2.00 (9)	ns^a^
	5–9	−17.86 ± 1.76 (8)	−15.47 ± 0.98 (10)
	10–15	−10.62 ± 1.06 (10)	−10.76 ± 1.30 (8)
	adult	−11.21 ± 0.80 (12)	−10.70 ± 0.66 (16)	ns^b^

*Values reflect means and standard errors, n: numbers of neurons, ns: statistically non-significant. Statistics, ^a^two-way ANOVA, ^b^Student *t*-test. The action potential threshold of poly I:C-treated group was lower than that of PBS treated-group in the neonatal period, but not in adulthood. There was no significant difference in action potential amplitude and afterhyperpolarization amplitude in any developmental period between PBS-treated and poly I:C-treated groups*.

**Table 2:** Summarized data of comparisons between offspring from PBS-treated and poly I:C-treated dams.

	Postnatal Day		PBS (*n*)	Poly I:C (*n*)	Statistics
sEPSCs frequency (Hz)	0–4		0.38 ± 0.10 (15)	0.23 ± 0.05 (15)	*F*_(1.88)_ = 4.72
					*P* < 0.05^a^
	5–9		0.42 ± 0.11 (20)	0.54 ± 0.08 (27)
	10–15		2.09 ± 0.28 (8)	1.30 ± 0.41 (9)
	adult		4.23 ± 1.50 (16)	1.07 ± 0.13 (16)	*P* = 0.054^b^
sEPSCs amplitude (pA)	0–4		9.90 ± 0.70 (15)	9.64 ± 0.41 (15)	*F*_(1.88)_ = 3.43
					*P* = 0.07^a^
	5–9		12.68 ± 1.06 (20)	11.34 ± 0.50 (27)
	10–15		15.05 ± 1.16 (8)	12.49 ± 1.37 (9)
	adult		14.10 ± 0.76 (16)	12.23 ± 0.29 (16)	*P* < 0.05^b^
mEPSCs frequency (Hz)	10–15		0.45 ± 0.08 (10)	0.59 ± 0.12 (14)	ns^b^
	adult		0.92 ± 0.07 (23)	0.67 ± 0.05 (24)	*P* < 0.01^b^
mEPSCs amplitude (pA)	10–15		10.65 ± 0.37 (10)	11.58 ± 0.45 (14)	ns^b^
	adult		12.89 ± 0.33 (23)	11.94 ± 0.37 (24)	*P* = 0.06^b^
sGABA-PSCs frequency (Hz)	0–4		1.16 ± 0.28 (18)	0.92 ± 0.21 (12)	ns^a^
	5–9		2.52 ± 0.57 (17)	2.65 ± 0.54 (16)
	10–15		4.64 ± 0.79 (19)	3.25 ± 0.42 (21)
	High	0–4	6.14 ± 0.94 (18)	5.74 ± 1.21(12)	*F*_(1.97)_ = 3.98
	K^+^	5–9	9.72 ± 1.12 (17)	14.06 ± 1.35 (16)	*P* < 0.05^a^
		10–15	12.56 ± 1.32 (19)	15.44 ± 1.77 (21)
	adult		5.30 ± 0.41 (13)	7.08 ± 0.57 (12)	*P* < 0.05^b^
sGABA-PSCs amplitude (pA)	0–4		26.47 ± 3.08 (18)	26.53 ± 4.07 (12)	ns^a^
	5–9		23.02 ± 1.97 (17)	20.00 ± 1.25 (16)
	10–15		21.95 ± 1.87 (19)	19.60 ± 1.18 (21)
	High K^+^	0–4	29.55 ± 2.76 (18)	32.90 ± 7.41 (12)	ns^a^
		5–9	35.93 ± 2.62 (17)	27.73 ± 2.04 (16)
		10–15	31.58 ± 3.05 (19)	29.14 ± 2.20 (21)
	adult		15.53 ± 0.86 (13)	18.47 ± 2.44 (12)	ns^b^
mGABA-PSCs frequency (Hz)	0–4		0.29 ± 0.06 (14)	0.66 ± 0.17 (11)	ns^a^
	5–9		0.88 ± 0.16 (14)	0.76 ± 0.17 (11)
	10–15		0.94 ± 0.14 (13)	1.14 ± 0.26 (15)
	adult		2.99 ± 0.41 (13)	3.05 ± 0.65 (10)	ns^b^
mGABA-PSCs amplitude (pA)	0–4		21.28 ± 2.07 (14)	21.96 ± 2.36 (11)	ns^a^
	5–9		18.43 ± 1.90 (14)	16.92 ± 1.54 (11)
	10–15		19.21 ± 1.84 (13)	16.02 ± 1.58 (15)
	adult		13.54 ± 1.54 (13)	14.41 ± 1.32 (10)	ns^b^

*Values reflect means and standard errors, n: numbers of neurons, ns: statistically non-significant. Statistics, ^a^two-way ANOVA, ^b^Student *t*-test*.

## Discussion

Several epidemiological studies have suggested that maternal infection in humans is a risk factor for schizophrenia and autism (Brown, 2006, 2012). Animal studies have suggested that prenatal maternal infection and maternal infection responses trigger neurodevelopmental deficits and induce behavioral abnormalities (Deverman and Patterson, 2009; Meyer, 2014). In our study, prenatal immune activation by poly I:C reduced excitatory synaptic inputs and increased inhibitory (GABAergic) drive onto hippocampal CA1 pyramidal cells in adulthood, which could result in net inhibition of the hippocampal CA1 neuronal circuit. We also observed that poly I:C treatment reduced excitatory drive and increased GABAergic drive in the early postnatal period, suggesting that the impact of prenatal immune activation on hippocampal neuronal circuits gradually emerges from the early postnatal period and becomes evident in adulthood.

Maternal immune activation in rodents has been shown to induce behavioral abnormalities relevant to schizophrenia (Meyer, 2014). In this study, we observed that prenatal poly I:C treatment reduced excitatory drive and excitatory synaptic inputs and increased inhibitory drive on pyramidal cells in the hippocampus of adult mice. Hippocampal lesions in adulthood enhance sensitivity to the PPI-disruptive effects of dopamine receptor agonists (Swerdlow et al., 2000). Hippocampal abnormalities are a pathophysiological substrate of schizophrenia (Heckers and Konradi, 2010). The alteration of excitatory and inhibitory synaptic transmission caused by prenatal poly I:C treatment in the adult hippocampus may underlie the behavioral abnormalities relevant to schizophrenia.

NVHLs have been shown to induce schizophrenia-like behavior. In NVHL animal models, dysfunction of the hippocampus during early neuronal development affects the development of other brain regions, including the prefrontal cortex, and induces abnormal behaviors in adulthood (Tseng et al., 2009). We observed that prenatal poly I:C treatment decreased excitatory drive and increased GABAergic drive onto CA1 pyramidal cells in the neonatal period. The inhibition of neonatal hippocampal glutamatergic synaptic transmission by poly I:C treatment may impact neuronal circuit development in other brain regions in animal models of NVHL. During the neonatal period, GABAergic signaling is thought to drive excitatory transmission (Ben-Ari, 2002). However, even if prenatal poly I:C treatment increases excitatory GABAergic signaling, it might not be able to compensate for the loss of glutamatergic excitatory transmission due to locally distributed GABAergic neuronal circuits. Conversely, adult hippocampal dysfunction caused by maternal immune activation could directly underlie abnormal behavior; neonatal hippocampal dysfunction may also underscore aberrant behaviors in this animal model of maternal immune activation.

In the present study, prenatal poly I:C treatment affected both spontaneous and miniature EPSCs, and spontaneous but not miniature GABA-PSCs in adulthood. Our interpretation is that prenatal poly I:C treatment affected only sGABA-PSCs because it affects the excitability of GABAergic interneurons, correlating with spontaneous spikes but not the number of synapses and the transmitter releasing mechanism. Future studies by recording from genetically-identified GABAergic interneurons as parvalbumin-positive interneuron, somatostatin positive interneuron, and so on are necessary to assess how prenatal poly I:C treatment affect the excitability of GABAergic interneurons. Alternatively, prenatal poly I:C treatment might affect both sEPSCs and mEPSCs, impacting upon both the excitability of and synaptic transmission from presynaptic pyramidal cells. Prenatal poly I:C treatment reduced sEPSC frequency, but not that of mEPSCs, in the early postnatal period. The treatment affected both sEPSC amplitude and mEPSC frequency in adulthood. Generally, the frequency of mEPSCs is influenced by transmitter release probability and the number of synapses. Also, the frequency of sEPSCs is influenced by presynaptic action potentials. Poly I:C treatment may primarily reduce the excitability of glutamatergic presynaptic neurons (e.g., CA3 pyramidal cells) in the early postnatal period; this activity-dependent effect may induce a reduction in functional synapses or transmitter release probability in adulthood. We found that the action potential threshold of the poly I:C-treated group was lower than that of the PBS-treated group in the neonatal period, but not in adulthood. Maternal immune activation has been shown to induce the expression of cytokines in the fetal brain (Arrode-Brusés and Brusés, 2012). These cytokines might increase hippocampal neuronal excitability in the neonatal period (Pineda et al., 2013). However, it remains unclear why the effects of poly I:C treatment on the excitability of CA1 pyramidal cells disappear in adulthood. Future studies are required to understand this question.

Several studies have reported that maternal immune activation by poly I:C and lipopolysaccharide decrease hippocampal excitatory synaptic transmission mainly expressed as field excitatory postsynaptic potential (fEPSP) in CA1 (Lowe et al., 2008; Ito et al., 2010; Oh-Nishi et al., 2010; Escobar et al., 2011; Patrich et al., 2016b), whereas in CA3 it increases sEPSC frequency and amplitude (Fernandez et al., 2019). Thus, maternal immune activation might have different effects on excitatory synaptic transmission in different areas of the hippocampus. In the present study, poly I:C treatment increased GABAergic synaptic transmission expressed as sGABA-PSC frequency. A possible explanation might be that the treatment increased glutamic acid decarboxylase (GAD67) expression (Harvey and Boksa, 2012; Cassella et al., 2016), which led to the need for increased GABA synthesis. Although we could not ascertain the effect of poly I:C treatment on mGABA-PSC, mIPSC has been reported to increase (Patrich et al., 2016a) as well as decrease (Ducharme et al., 2012). These differences might result from either the difference between acute and cultured slices or from the timing of poly I:C administration.

We previously reported that prenatal poly I:C treatment delayed hippocampal myelination in the early postnatal period (PD14; Makinodan et al., 2008). In the present study, prenatal poly I:C treatment reduced glutamatergic synaptic inputs onto CA1 pyramidal cells during PD0–PD15. Release of glutamate from synaptic vesicles along axons promotes myelination induction (Wake et al., 2011). If poly I:C treatment-induced reduction of glutamatergic synaptic transmission appears in CA1 pyramidal cells as well as axons from CA3 to CA1, reduced glutamate release in the neonatal period may cause delayed myelination, following our previous findings.

In this study, we found that maternal immune activation reduced excitatory transmission and increased GABAergic (inhibitory) transmission. However, excitatory transmission and GABAergic (inhibitory) transmission were measured during different recordings under different conditions. To accurately assess the balance of excitation/inhibition transmission, it is preferable to use a standard protocol (Zhou et al., 2009; Widman and McMahon, 2018) to assess both synaptic transmissions simultaneously in the same neuron. In the future, studies using the standard protocol are necessary to assess the effect of maternal immune activation on E/I balance.

## Data Availability Statement

All datasets presented in this study are included in the article.

## Ethics Statement

The animal study was reviewed and approved by The animal care and use committee of Nara Medical University.

## Author Contributions

KN, YO, and HY designed the research and performed the experiments. KN, HY, KY, and SK analyzed the data. HY and KN wrote the article. YN, MM, MY, YS, and TK supervised the project.

## Conflict of Interest

The authors declare that the research was conducted in the absence of any commercial or financial relationships that could be construed as a potential conflict of interest.

## Abbreviations

ACSF: artificial cerebrospinal fluid
CA: cornu ammonis
CNQX: 6-cyano-7-nitroquinoxaline-2,3dione
ED: embryonic day
fEPSP: field excitatory postsynaptic potential
MBP: myelin basic protein
mGABA_A_-PSCs: miniature GABA_A_-postsynaptic currents
NVHL: neonatal ventral hippocampal lesions
PBS: phosphate-buffered saline
PD: postnatal day
poly I:C: polyriboinosinic-polyribocytidilic acid
PPI: prepulse inhibition
PSC: postsynaptic currents
sEPSCs: spontaneous excitatory postsynaptic currents
sGABA_A_-PSCs: spontaneous GABA_A_ receptor-mediated postsynaptic currents
TTX: tetrodotoxin.
